# Development of brain organoid technology derived from iPSC for the neurodegenerative disease modelling: a glance through

**DOI:** 10.3389/fnmol.2023.1173433

**Published:** 2023-08-03

**Authors:** Amirah Syamimi Jusop, Kalaiselvaan Thanaskody, Gee Jun Tye, Sylvia Annabel Dass, Wan Safwani Wan Kamarul Zaman, Fazlina Nordin

**Affiliations:** ^1^Centre for Tissue Engineering and Regenerative Medicine (CTERM), Faculty of Medicine, Universiti Kebangsaan Malaysia, Kuala Lumpur, Malaysia; ^2^Institute for Research in Molecular Medicine (INFORMM), Universiti Sains Malaysia, Minden, Penang, Malaysia; ^3^Department of Biomedical Engineering, Faculty of Engineering, Universiti Malaya, Kuala Lumpur, Malaysia

**Keywords:** neurodegenerative disease, induced pluripotent stem cells (iPSCs), disease modeling, brain organoids, Alzheimer’s disease, assembloids

## Abstract

Neurodegenerative diseases are adult-onset neurological conditions that are notoriously difficult to model for drug discovery and development because most models are unable to accurately recapitulate pathology in disease-relevant cells, making it extremely difficult to explore the potential mechanisms underlying neurodegenerative diseases. Therefore, alternative models of human or animal cells have been developed to bridge the gap and allow the impact of new therapeutic strategies to be anticipated more accurately by trying to mimic neuronal and glial cell interactions and many more mechanisms. In tandem with the emergence of human-induced pluripotent stem cells which were first generated in 2007, the accessibility to human-induced pluripotent stem cells (hiPSC) derived from patients can be differentiated into disease-relevant neurons, providing an unrivaled platform for *in vitro* modeling, drug testing, and therapeutic strategy development. The recent development of three-dimensional (3D) brain organoids derived from iPSCs as the best alternative models for the study of the pathological features of neurodegenerative diseases. This review highlights the overview of current iPSC-based disease modeling and recent advances in the development of iPSC models that incorporate neurodegenerative diseases. In addition, a summary of the existing brain organoid-based disease modeling of Alzheimer’s disease was presented. We have also discussed the current methodologies of regional specific brain organoids modeled, its potential applications, emphasizing brain organoids as a promising platform for the modeling of patient-specific diseases, the development of personalized therapies, and contributing to the design of ongoing or future clinical trials on organoid technologies.

## 1. Introduction

Neurodegenerative diseases (ND) are immediate and debilitating disorders characterized by relentless progressive atrophy of neuronal pathology that results in impaired ataxia and cognition, leading to death (Wu et al., 2019). There are a number of neurodegenerative diseases, comprises of Huntington’s disease (HD), Alzheimer’s disease (AD), Amyotrophic Lateral Sclerosis (ALS), Parkinson’s disease (PD), Spine Cerebral Atrophy (SCA), and Spinal Musculosis Atrophy (SMA), which affect the elderly and are common age-related diseases (Martier and Konstantinova, 2020). In addition to advancing age, the greatest known risk factors for ND are genetic defects (Hou et al., 2019). Numerous studies have elucidated the molecular mechanisms that contribute to the etiology and pathogenesis of these diseases. Among the proposed factors contributing to ND are degeneration and decomposition of defective proteins, the production of oxidative stress and free radicals, mitochondrial impairment, a large number of decomposition mechanisms by proteases, and metal dehydrostatics (Yousefi et al., 2020).

Many ND do not have successful treatments due to the limited access of cells to observe recapitulation of physiological and pathological mechanisms of the disease (Camnasio et al., 2012). ND have complicated pathogenic pathways that are still mostly unexplored. Most human cell lines and animal models are created with artificial methods or strategies of genetic overexpression, which may not accurately reflect the pathophysiology of human disease furthermore, there is no clear consensus on the designation of these new structures, making it difficult for researchers and the public to follow and clearly define technological advances along with needs (Pașca et al., 2022). In addition, the human central nervous system is very different from the normal laboratory animal, making primate testing impractical. This leads to the incapacity to establish suitable therapeutic approaches to delay the occurrence or cure the development of ND.

Decades after decades of research and experimentation on stem cells have been done, capitalizing on the advantage of their remarkable ability to divide and self-renew into undifferentiated cells. These special characteristics of pluripotent stem cells offer various prospects for different applications starting from the use of stem cells to understand model diseases, cell therapies, regenerative medicine, toxicological tests, etc. (Spitalieri et al., 2016). iPSCs are artificial pluripotent stem cells that are produced from somatic cells with the help of several pluripotency marker gene which then enable them to be differentiate into three primary germ layers which is ectoderm, endoderm, and mesoderm. Furthermore, iPSCs are similar to ESCs in aspects of proliferation and differentiation; however, it gives rise to the ethical limitation and legally banned in certain countries (Moradi et al., 2019; Bai, 2020). The last decade has witnessed the emergence of the first human-induced pluripotent stem cell (iPSC) technology by Takahashi and Yamanaka (2006) when they were able to reprogram the gene expression of the fibroblast genome by adding four pluripotency markers, OCT4, SOX2, KLF4, and C-MYC which then were known as Yamanaka factors (Takahashi and Yamanaka, 2006).

After these significant findings by Yamanaka team, many other researchers developed alternative methods of cellular reprogramming. There are various approaches to cell reprogramming that include viral transduction of specific transcription factors into somatic cells and efficiently drive the integration of reprogramming factors into selected somatic cells (Spitalieri et al., 2016). However, efficient delivery of pluripotency markers depends on the type of cells, conditioned culture medium, and suitable expression factors (Bahmad et al., 2017). According to Spitalieri et al. (2016), non-integration of viral reprogramming techniques, such as protein transmission, non-integration of viral vectors, such as Sendai viruses, episomal vectors, and transmission of modified mRNA transcripts, can avoid transgene integration, minimize risk of reactivation of oncogenes, insertion mutations, immunogenetic, reactivation of reprogramming genes, and avoid integrating systems approaches, leading to serious problems in the generation of iPSC. In a sense, the option of reprogramming methods really depends on the purpose of the research whether the objectives are focusing on the mechanisms of reprogramming or to generate the clinical grade of iPSC which subsequently will be used for disease modeling, cell therapy, or drug toxicity.

To illustrate, any methods of retroviral or transduction optimization in human fibroblasts and subsequent culture conditions can generate induced pluripotent stem cells (iPSCs) from adult human somatic cells. These efforts have enabled us to generate iPSCs from adult human dermal cell cells and other human somatic cells, such as hematopoietic stem cells, adipocytes and peripheral blood mononuclear cells, with a differentiation potential comparable to that of human embryonic stem cells *in vitro* and *in vivo* (Takahashi et al., 2007). By delivering an almost infinite amount of pluripotent material from any patient, iPSC technology has offered new opportunities to uncover disease-modulating treatments plus the genomes of iPSCs can be edited to introduce or correct disease-associated variants (Pantazis et al., 2022). In addition, iPSCs have proved to be significant in various areas of studies in virology, including modeling target organ viruses such as Cerebral Malaria (CM), HIV and SARS – CoV-2, toxicology, and doubtless in the modeling of neurological diseases, using which has established innovative opportunities for both mechanistic types of research and recognition of new disease treatments (di Val Cervo et al., 2021; Ostermann and Schaal, 2023; Silva-Pedrosa et al., 2023a).

**Figure 1:**
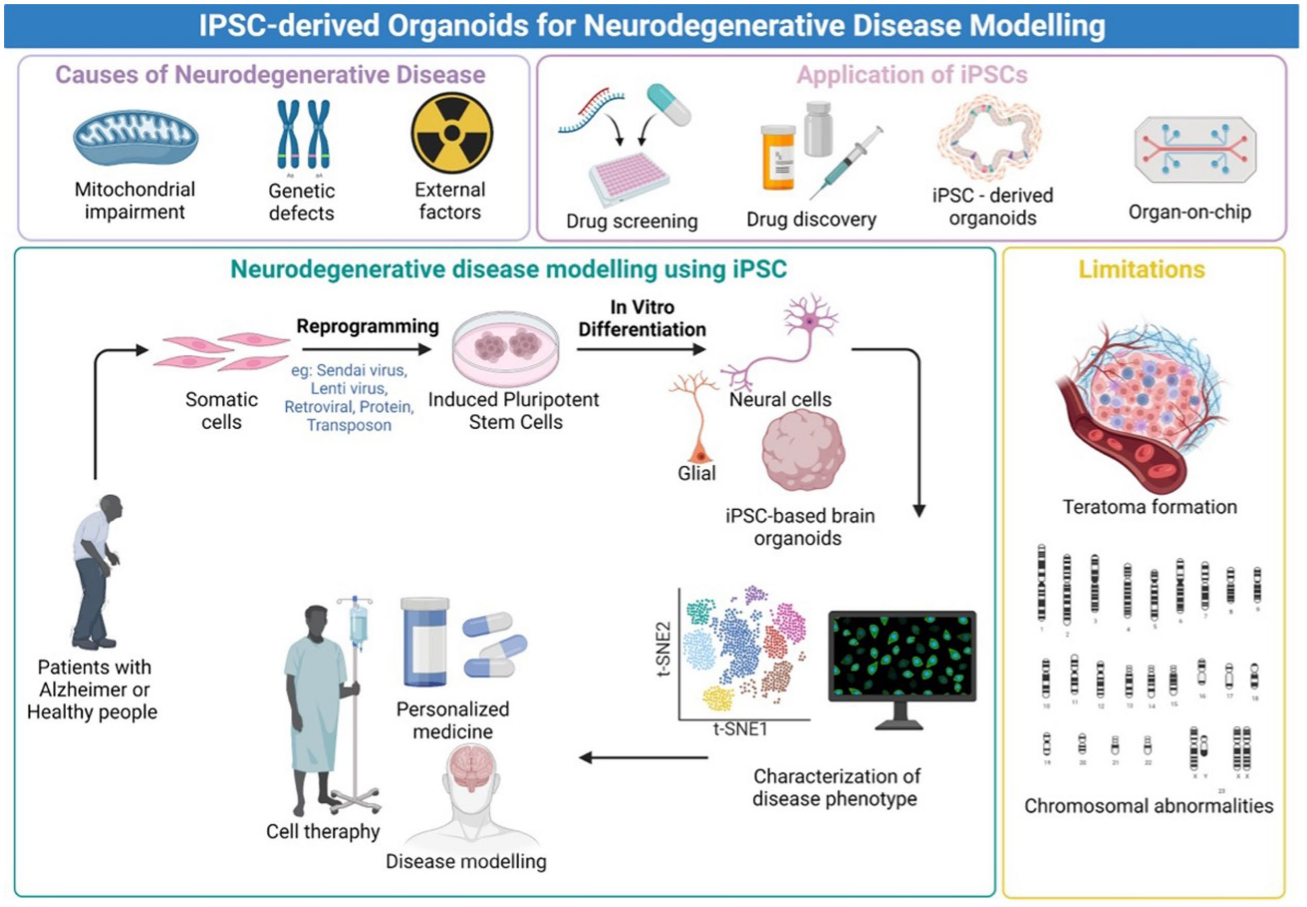
Graphical Abstract. An overview of the iPSC-derived organoids for neurodegenerative disease modeling. Created with Biorender.com.

Following this unprecedented discovery of the potential to replicate patient somatic cells to iPSCs, the platform provides an advanced predictive modeling platform for creating disease-related cells for *in vitro* disease modeling. Furthermore, the emergence of three-dimensional (3D) neural tissue from the iPSC called brain organoid has provided a major insight into the modeling of neurological diseases (Mansour et al., 2018). Three-dimensional (3D) models, such as brain organoids made from iPSCs, assembloids, and grafted organoids, have recently come into existence, and they may be useful for studying the pathogenic characteristics of neurodegenerative illnesses as being summarized in Figure 1. The human brain organoid needs to be self-assembled by stem cells and different types of different cells, like the unique structure of the actual human brain region, to simulate specific changes in neurological disorders for disease modeling. Organoids may be combined to create assembloids, which allow for the functional modeling of processes including neuro-immune interactions, cell migration, and circuit building (Hong and Do, 2019; Pașca et al., 2022).

This review paper aims to provide a comprehensive overview of recent discoveries in brain organoids-based modeling of neurodegenerative diseases, especially Alzheimer’s disease (AD), which have been established or are currently in the process of being generated, as well as to highlight relevant research areas for the future. Furthermore, we reviewed recent advances in iPSC-based organoids and other platforms for disease modeling and pointed out some key hurdles in specialized cell reprogramming. Finally, the important applications of iPSCs have been discussed with a special emphasis on organoids derived from iPSCs as promising and intriguing sources for modeling patient-specific diseases and developing personalized therapies.

## 2. iPSCs offer great therapeutic potentials for neurodegenerative diseases

Recent developments in stem cell research have contributed to the production of iPSCs, which are specifically effective in the treatment of neurodegenerative illnesses. iPSCs, in particular, by providing infinite sources of stem cells, we bypassed the ethical limitations of the human embryonic stem cells that were obtained from blastocyst and interrupted the development of a viable embryo (Bordoni et al., 2018). As such, iPSCs have been featured in various applications, including transplantation to treat macular degeneration, corneal transplants, heart failure, diabetes, immunotherapy, and open new approaches in modeling the pathogenesis of the disease of the central nervous system (Bordoni et al., 2018; Chang et al., 2020). iPSCs have emerged as a prominent alternative to embryonic stem cells in clinical settings and promise great potential to understand disease mechanisms and contribute much to drug screening. One of the advantages of using iPSC is that patients’ derived iPSCs can be used without the constraints of limited donor cell accessibility to study patient disease (Hong and Do, 2019). The reason being that iPSCs can specifically be differentiated efficiently from individual patient cells from the ectoderm, endoderm, or mesoderm lineage and thus exhibit the unique patient’s genetic background (Abdullah et al., 2012; Marotta et al., 2020). Most significantly, they are ideal for disease modeling and regenerative medicine due to their pluripotency and self-renewal properties.

**Figure 2:**
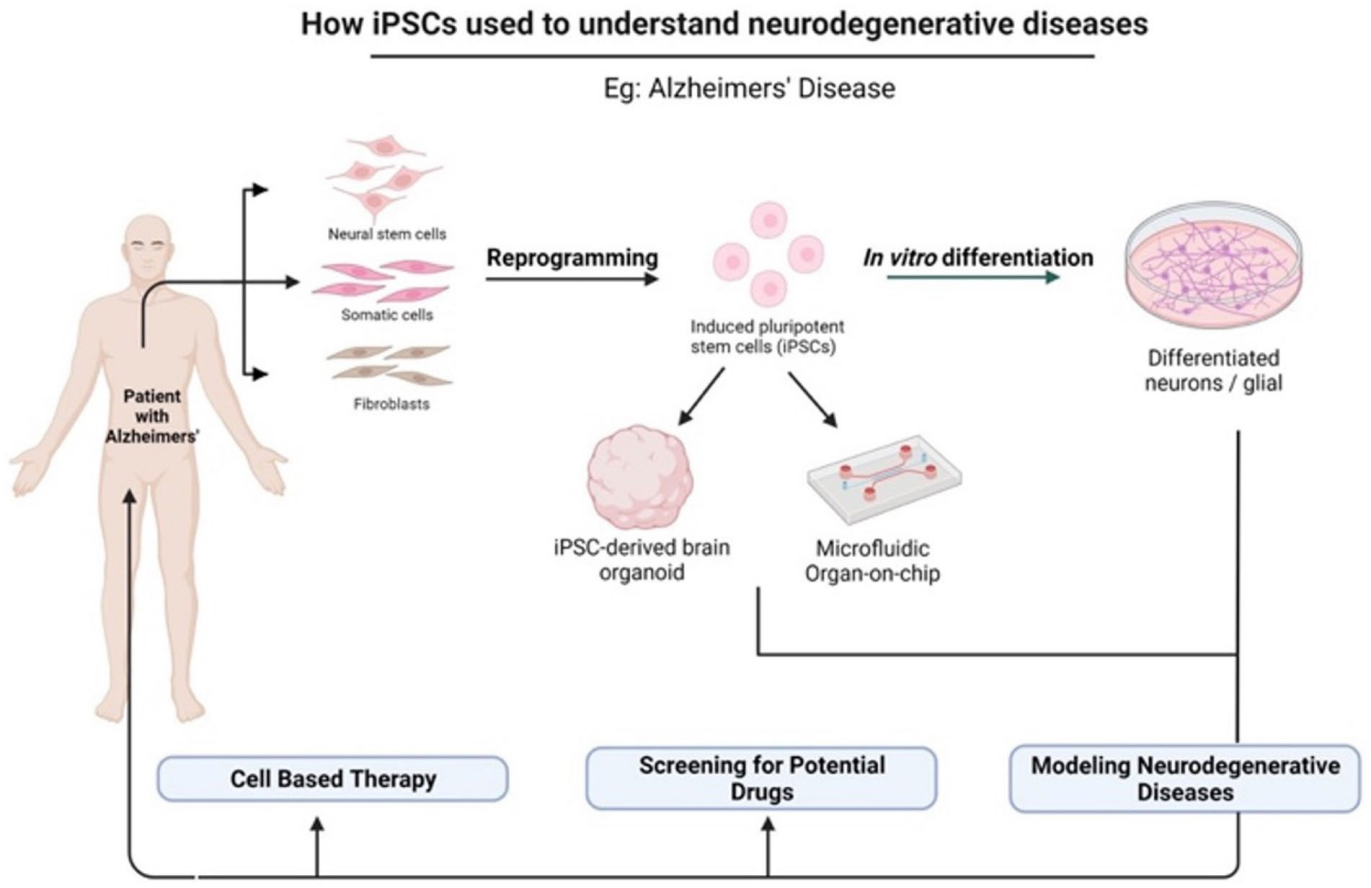
The potential application of iPSCs in understanding neurodegenerative diseases. iPSCs can be reprogrammed from various somatic cells and differentiated into multiple cell lineages because they possess unique properties of self-renewal and pluripotency. There are many applications of iPSCs in the fields of gene therapy, disease modeling, and drug discovery. Created with Biorender.com.

**Figure 3:**
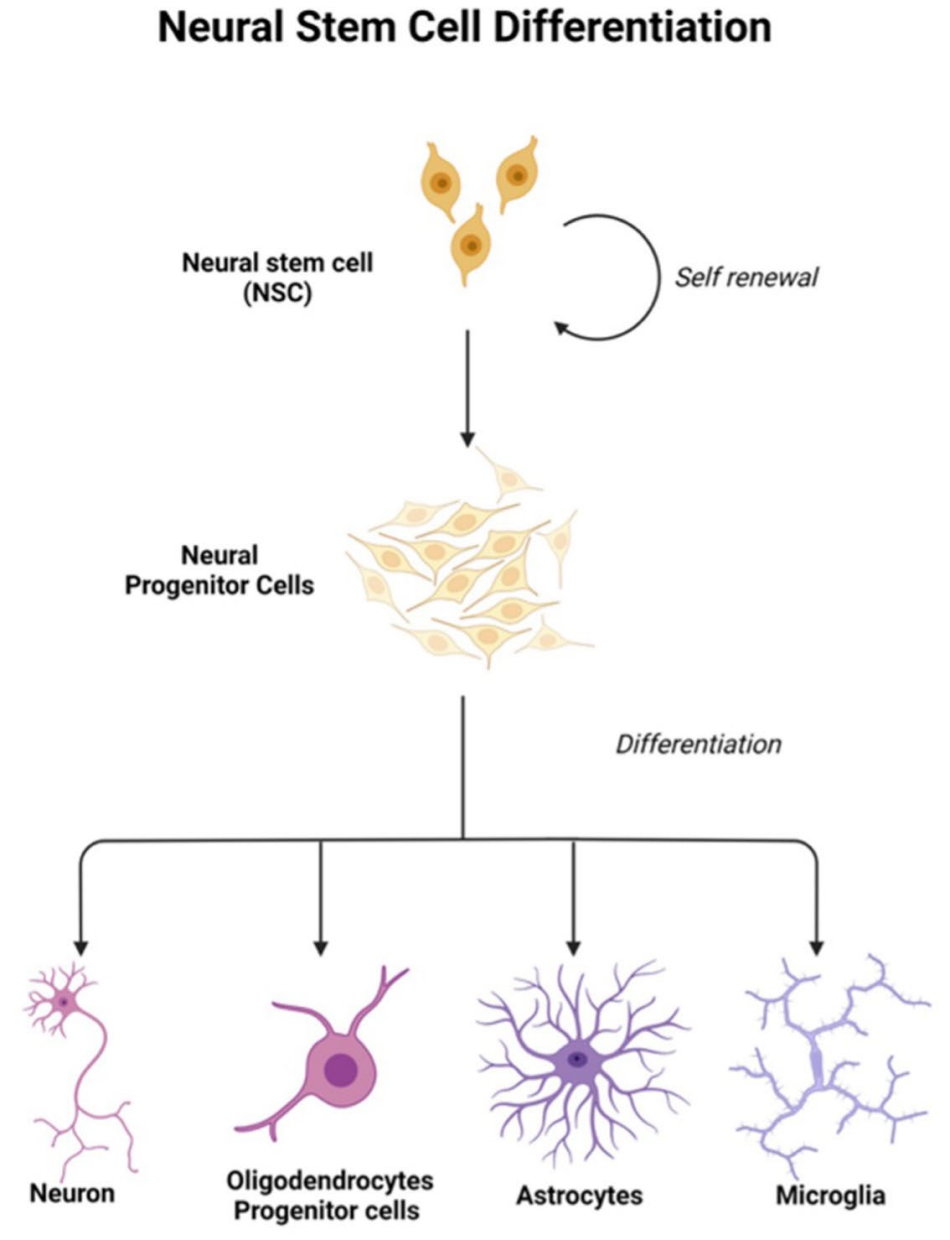
Neural stem cells are tripotent cells that can differentiate into neural lineage cell subtypes like neurons, oligodendrocytes, and astrocytes. Created with Biorender.com.

In light of the limits of researching the human brain, iPSC technology has recently been the subject of substantial research for neurological illnesses. Due to the direct generation of iPSCs from neurological illness patients, 2D and 3D models may be used to research nervous system disorders *in vitro* (Silva-Pedrosa et al., 2023b). Currently, iPSCs and neural stem cell cultures allow cell reprogramming and differentiation into neurones and glial cells (astrocytes, oligodendrocytes, and microglia) that contribute to the modeling of neurodegenerative diseases as described in Figure 2; Korhonen et al., 2018). Most neural cells, such as neurones, astrocytes, oligodendrocytes, and microglia, can be used to generate iPSCs by implementing a different method of reprogramming and type of pluripotency factors used, as different starting cell lines exhibit different properties and the protocol must be modified to suit each research capacity (Abud et al., 2017; Hong et al., 2022). Figure 3; Table 1 described the application of different types of neuron cells that can be differentiated from iPSCs.

**Table 1:** Type of neurone cells that can be derived from iPSC.

*Cell Types*	Differentiated from	Objectives	Impact	References
Neural stem cells	Brain samples from COVID-19 patients	To investigate the susceptibility of hiPSC-derived brain cells and their organoids to SARS-CoV-2 infection.	SARS-CoV-2 only infects neurones and astrocytes, but epithelial cells of the epithelial cells of the choroid plexus underwent robust infection.	Jacob et al. (2020)
Neurons	Patient Fibroblast	To reveal the hyperexcitability of motor neurones derived from ALS patients.	Motor neurones derived from iPSC from ALS patients are hyperexcitable compared to controls.	Wainger et al. (2014)
Astrocytes	Neurones from PD patients.	To study the pathogenesis of PD from patient-specific iPSC-derived astrocytes.	Astrocytes contribute to dopaminergic neurodegeneration in PD.	di Domenico et al. (2019)
Microglia	Mouse brain	To understand the complex properties of human microglia.	The combination of hiPSC-based 3D culture and human-mouse microglial chimeric brain models contributes to the study of human microglia and its pathogenesis of neurological disorders.	Jiang et al. (2020)
Oligodendrocytes Progenitor Cells (OPC)	4 different cell lines: WA09/H9 hESCs and K04, C14, and C27 iPSCs	Establish potential strategies for cell-based repair of demyelinated brain and spinal cord lesions that can myelinate and rescue a mouse model of congenital hypomyelination.	HiPSCs-OPC have higher speed and efficiency of myelination compared to fetal tissue-derived OPC with no tumors observed up to 9 months after transplantation to treat patients with myelin loss disorders.	Wang et al. (2013)
Dopaminergic neurones	Monkey neural cells	To analyze iPSC-derived midbrain dopamine neurones from cynomolgus monkey (CM) for up to 2 years following autologous transplantation in a Parkinson’s disease (PD) model.	Data show neurologically relevant functional improvements with concomitant positive neuroimaging with strong immunological, functional, and biological rationale for using iPSC midbrain dopamine neurones.	Swistowski et al. (2010), Hallett et al. (2015)

Even tough iPSC-based therapies may take some times to become clinical treatments despite current limitations that require the provision of sophisticated models to thoroughly study the intrinsic factors linked to neurodegenerative disease. By reviewing important features of these disorders, iPSCs are frequently employed to comprehend the mechanism of neurodegenerative diseases. Researchers may produce precise models precisely using patient cells by employing these methods. A model of Parkinson’s disease (PD) was created from early Parkinson’s disease patients with young PSCs, which confirmed the implication of lysosomal degradation pathways, mitochondrial dysfunction, and mitophagy impairment in pathogenesis (Valadez-Barba et al., 2020). The other example of different diseases is that Penney et al. (2020) have highlighted the iPSC-based model for studying Alzheimer’s disease (AD). Most AD studies will incorporate mutated genes that cause AD such as APPV717I, PSEN1, PSEN2, and APOE4 which potentially arises from defective mitophagy, mitochondrial dysfunction, elevated oxidative stress and oxidative damage that affects the functional properties of mature neurons in AD patients (Penney et al., 2020). Most characteristics can be validated by the establishment of patient-specific iPSC-derived models hence will facilitate unfindable cure for the neurodegenerative disease.

Personalized regenerative medicine using pluripotent stem cells offers impressive potential for numerous iPSC-derived therapeutics, however, the inherent uncertainty of first-in-human experiments and the technical complexity of multipotent stem cells make early multipotent stem cell experiments more ethically difficult. Early clinical trials should not involve iPSC transplantation in humans in the early stage. Instead, to show a great strength of iPSC derived from patient-specific, they should create the way for personalized therapies and assessment in clinical purposes. Clinical trials have been initiated with cellular therapeutic products derived from human iPSC, and are currently being evaluated for effectiveness and safety, although clinical trials with human multipotent stem cells are in the early stages (Doss and Sachinidis, 2019). Table 2 shows key issues in preclinical studies for neurological diseases. On top of that, only 6 years after the publication of Takahashi and Yamanaka’s seminal article showing the derivation of iPSCs from adult human fibroblasts, the first trial using iPSCs was announced for a study in patients with macular degeneration, a common cause of blindness (Habets et al., 2014). Although the field of iPSCs has made significant progress, there are still inherent limitations that must be critically addressed if hope for the effective clinical utility of these cells in regenerative medicine is to become a reality.

**Table 2:** Key issues in preclinical studies for iPSC-based neurological diseases.

Diseases	Year and status	Country	Cells associated	Primary objectives	Remarks	References
Parkinson’s disease	07 September 2017	Japan	Dopaminergic neurons in the midbrain.	To address the efficacy and safety of human iPSC-derived dopaminergic neurons function in a primate.	The longest preclinical trial with the largest number of monkeys A simulation of the planned clinical trial in 2018.	Kikuchi et al. (2017)
Parkinson’s disease	1 August 2018 (Ongoing)	Japan	Dopaminergic progenitors derived from iPSC.	To observe the incidence and severity of adverse events and the presence or absence of graft expansion in the brain 24 months after transplantation.	First clinical trial using human iPSCs Patients in the middle stage of PD were chosen for the clinical trial because in the severe stage of PD, the striatal neurons and innervating cortical neurons have already degenerate.	Kikuchi et al. (2017)
Spinal cord injury	March 13, 2019 (Ongoing)	Japan	neural stem/progenitor cell grafts (iPSC) derived from neural stem/progenitor cells (hiPSC-NS/PC).	Assess the safety of hiPSC-NS/PC transplantation in patients with subacute SCI.	They plan to start recruiting a patient as soon as the COVID-19 epidemic subsides.	Sugai et al. (2021)

Consequently, the annotation for clinical trials in neurodegenerative diseases in accordance with the World Health Organization’s International Clinical Trials Registry Platform (ICTRP) and clinicaltrials.gov databases, we identified that there are 112 clinical trials available upon filtration using the keyword “iPSC” and “induced pluripotent stem cells” from Clinical Trials website. Based on the ICTRP, 31 trials were initially identified, but 13 projects were repeated in the results and therefore eliminated, leaving 18 trials for further analysis. Based on the study published in 2020, they have summarized that noncommunicable diseases such as neurological disorders only account for 12.9% of other diseases such as cardiovascular, musculoskeletal, and sense organ diseases (Deinsberger et al., 2020). Notwithstanding all the advantages of iPSCs, various safety and ethical issues should be considered, as the failure of premature trials can jeopardize the safety of participants and inhibit the development of essential therapeutics, as in early genetic therapy trials, due to social riots.

## 3. iPSC-based organoids of neurological diseases

**Figure 4:**
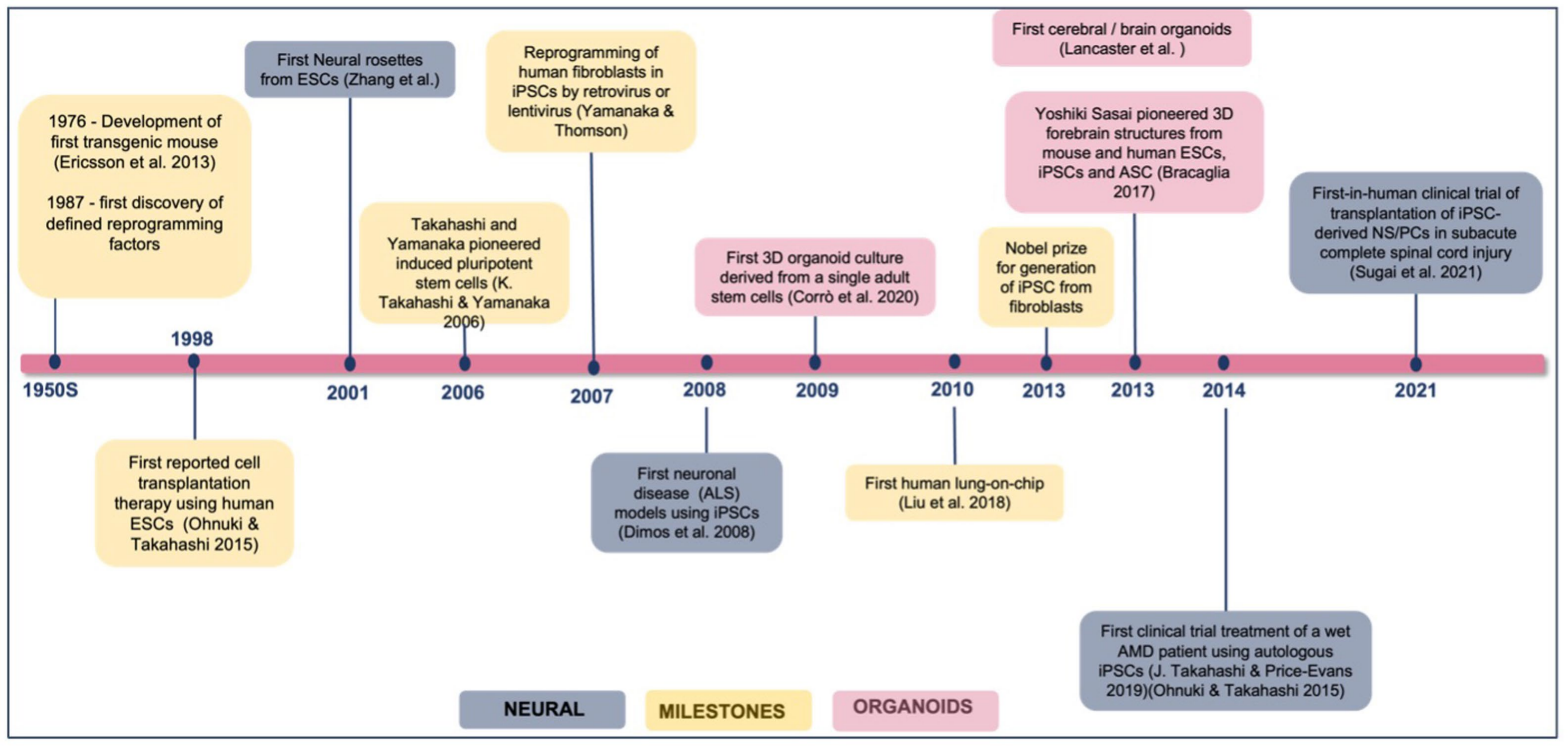
Achievements and milestones in iPSC research. Timeline showing the achievements of iPSC technology since 1976, divided into general milestones and time points when neural derivatives were first successfully generated.

iPSC for disease modeling and drug screening is a field that is still developing and Figure 4 shows Yamanaka and colleagues’ development of iPSC and use of three-dimensional organoids, tissues engineering, microfluidic organ chips, and humanized animal systems to create more sophisticated iPSC-based systems. Various new technologies may be integrated or utilized in parallel with iPSC models to improve *in vitro* disease models and drug screening platforms (Rowe and Daley, 2019). Elucidating the biological basis of diseases of the nervous system remains an extensive scientific challenge. Therapeutic development of human neural connections requires a thorough understanding of its mechanisms of development and functioning, which is difficult due to the lack of accessibility that perfectly mimics human brain material and *in vitro* models of lineage-specific connection (Sarkar et al., 2018). Furthermore, due to the interaction of genetic predisposition, developmental history, psychological factors, and environmental exposures, the symptoms and severity of neuropsychiatric illnesses vary greatly between individuals (Amin and Paşca, 2018). A range of models has been developed to investigate brain disorders, each model carrying its advantages and limitations as listed in Table 3. This table shows comparison between iPSC-derived multilineage platforms that have grown in complexity from simple, two-dimensional cultures to engineered platforms and complex organoids since the discovery of iPSCs in 2007 and most significantly contribute to the recapitulation of disease mechanism and its pathophysiology.

**Table 3:** Differences between multicell lineage model platform for disease modeling.

Multi-lineage platform	Study area	Advantages	Limitations	References
2D monolayer culture	To study metabolism disease	Highly scalable and acceptable for quick experiments to observe cellular metabolism, transcriptional, expression, genetic variability, and signaling mechanism.	Lack of *in vivo* tissue microarchitecture and physiological functionality.	Sharma et al. (2020), Collin de l’Hortet et al. (2019)
3D Microfluidic Engineering and organ-chip technology	To mimic individual organ functions for disease modeling and drug screening	Mimics the cellular microenvironment such as permitting tissue and organ functionality by recreating multicellular architectures, tissue-tissue interfaces, mechanical forces, physiochemical microenvironments, and vascular perfusion.	Expensive and modest scalability need to standardize for large-scale production of devices for the market.	Vatine et al. (2019), Ma et al. (2021)
Animal model and Chimera	For preclinical testing of medications	Physiologically relevant *in vivo* platform to study development and potentially serving as a system for mass-producing human tissues.	Low sensitivity, low throughput, and high cost. Not fully recapitulating human organ function.	Pei et al. (2016)
Organoid	To resemble *in vivo*-derived tissue	More realistically mimic *in vivo* developmental processes and settings.	Difficult to grow, time-consuming, and labor intensive. Lack multiscale architecture and tissue-tissue interfaces;	Ouchi et al. (2019), Bhatia and Ingber (2014)
Tissue Engineering and Bioprinting	To replicate the 3D complexity of cells and tissues	Mimic the mechanical and functional cues of the ECM, provide necessary stimuli, biochemical, and physical interactions.	Cost can be a limiting factor, biocompatible bioinks need to be refined.	Potjewyd et al. (2018)

In general, animal models such as rodents are indispensable tools for disease modeling that facilitate etiology and progression, discover associated path mechanisms, and validate therapeutic interventions, thus guiding the design of human clinical trials (Götz et al., 2018). Finding by Sharma et al. (2020) described the generation of three-dimensional organoids, microfluidic organ-chips, tissue engineering, and humanized animal systems have revolutionized the multilineage platforms and can recapitulate a variety of disease phenotypes thus contributing to drug discovery and disease modeling. The development of organoids derived from this have provided new insight and have contributed to the disease modeling of iPSCs (Sharma et al., 2020).

Organoids are defined as 3D multicellular aggregates derived from stem cells that differentiate and self-organize to recapitulate some aspects of normal and pathological processes for almost any human tissues (Rowe and Daley, 2019; Ho et al., 2022). Embryonic stem cells, induced pluripotent stem cells, neonatal or adult stem cells are typically used to derive these organoids (Corrò et al., 2020). Based on the current reported study, organoids derived from iPSCs have paved the way of stem cell research since they offer the possibility to combine the potential for self-organization of iPSCs and the possibility of potentially differentiate these cells to any organ-specific differentiation in comparison to organoids derived from adult tissue as claimed by Turhan et al. (2021). Brain development for *in vitro* models can also possibly established with the advent of iPSC-derived organoids technology with human brain being contemplated as the best resource for analyzing the mechanisms and pathophysiology of neuronal diseases (Matsui et al., 2020).

The brain organoids offer new systems that can overcome most of the hindrances and are constantly refined progressively. The self-organization of brain organoids in a three-dimensional environment may efficiently mimic human brain architecture and functions, such as differentiation (García-Delgado et al., 2022), mediate synaptic functions for brain circuitry development (Yakoub and Sadek, 2019) neuronal cell migration (Saglam-Metiner et al., 2023), and human-specific cell–cell and cell–matrix interactions (Muñiz et al., 2023) which facilitates human brain developmental morphogenesis. Thus, brain organoids may shed light on the process of neural tissue development and differentiation. Numerous studies support the potential of human brain organoids that have been summarized by Amin and Paşca (2018). They emphasized a few models of neurodevelopmental disorders such as congenital lissencephaly (Ji et al., 2023), brain folding disorders (Scott and Huang, 2022), Seckel syndrome (Ichisima et al., 2019), ischemia (Wang et al., 2020), etc. Ultimately, understanding complexity of nervous systems diseases and pathophysiological of human brains remain challenging despite current progress in neuroscience research. Nonetheless, a limitation barrier to apply the brain organoids as a treatment of neurodegenerative diseases resolve invasiveness in obtaining brain cells donor or human life brains and serve as one of powerful *in vitro* alternatives and hope to accelerate the neurodegenerative disease modeling (Li et al., 2023).

## 4. Brain organoids: type of specific regional brain and its methodologies

The methods used for generating human brain organoid which is extensively summarized by Matsui et al. (2020) are divided into two distinct categories (Matsui et al., 2020). Commonly, there are two different types of methodologies can be used to generate brain organoids: unguided methods and guided methods (Qian et al., 2019). The first protocol is unguided methods where to generate the brain organoid by differentiating iPSC through intrinsic signals, for instance, the induction of glutamatergic excitatory neurones and GABAergic inhibitory neurones without additional growth factor such as GDNF, NT-3. In 2013, a breakthrough discovery of organogenesis from stem cells in 3D culture by Yoshiki Sasai’s group where they first demonstrated guided methods, which are a series of polarized 3D differentiation protocols based on the serum-free culture condition and extrinsic signals to dictate the cell fate by inducing specific component of brain tissue and differentiate fundamental neural structures (Sasai, 2013; Mariani and Vaccarino, 2019) or otherwise, the protocol requires additional growth factors (Qian et al., 2019; Susaimanickam et al., 2022).

Brain organoids can be differentiated into cerebral organoids and region-specific brain organoids. Cerebral organoid derived from an unguided neural induction technique comprise independent and distinct tissues comparable to the brain area (i.e., not unique to the region; Lu et al., 2022). They mostly exhibit well-organized apical-basal polarity, neuronal migration, and functional maturation in 3D organoid tissues, which are morphological and functional parallels to the human growing brain cortex (Lancaster et al., 2013). While waiting for the development of the human cortex, ventral telencephalon, optic cup, thalamus, hypothalamus, midbrain, choroid plexus, striatum, etc. to be fully modeled, particular regional brain organs have been developed (Lu et al., 2022). Due to their greater relevance to the ND disease being modeled, region-specific brain organoids are being used more frequently. Regionally specific brain organoids may be produced repeatedly across different PS lines with consistency and homogeneity. Depending on the scientific topic one wishes to answer, one may select cerebral organoids or region-specific brain organoids. The creation of next-generation brain organoids by the assembly of complex brain areas with various region-specific brain organoids using updated procedures and bioengineering approaches will ideally lead to a better knowledge of ND disease processes and its treatment (Susaimanickam et al., 2022; Kim et al., 2023).

Most typical processes to create brain organoids derived from iPSCs are firstly self-aggregating the iPSC into embryoid bodies (EB) which then will be embedded the growing organoids in supporting extracellular matrix (Matrigel) inside the appropriate medium supplemented with specific growth factors under agitating conditions (Hopkins et al., 2021). Eigenhuis et al. (2023) had recently published the simplified and optimized protocols to generate robust dorsal forebrain organoids from iPSC-neural induction. They demonstrated an effective strategy by bypassing the initial steps which are embryoid body formation, neural induction, and cortical differentiation that are time-consuming into a new approaches which lessen the uses of media supplements and Matrigel (Eigenhuis et al., 2023).

Originally, first novel approaches in generating of whole cerebral organoid was reported initiated by Lancaster et al. (2013) where the differentiated hiPSCs of patient skin fibroblasts using lentiviral delivery of the four well-described reprogramming factors: Oct4, Sox2, c-Myc, and Klf4. These hiPSCs underwent neuroectoderm differentiation, mimicking an early stage of the human embryonic cerebral cortex’s development before maturing to form distinct pyramidal identities with minimal geographical separation. They then use patient-derived iPSCs and shRNA in these organoids to recreate the challenging to reproduce in mice CDK5RAP2 dependent etiology of microcephaly (Lancaster et al., 2013).

After all, it is also commonly acknowledged that the connections between various brain systems or areas play a crucial role in the development of neurodegenerative or psychiatric illnesses. Thus, the use of assembloids, which were created by physically joining various organoids, would encourage the investigation of therapeutic targets (Zhou et al., 2023). Recently, more advanced brain organoid systems have been created. The next generation of brain organoids may replicate varied interactions between different human brain areas in a dish by fusing or constructing separate region-specific brain organoids (Makrygianni and Chrousos, 2021). These specific systems, which are also known as assembloids, are capable of simulating directed cell migration and axonal projection in the growing human brain, including the tangential migration of human interneurons from the ventral to the dorsal forebrain, cortical glutamatergic neurons, and GABAergic interneurons (Makrygianni and Chrousos, 2021; Lu et al., 2022). However, more research is necessary to determine whether assembloids can accurately capture more subtle inter-regional changes linked to so-called connectopathies and to reliably assess connectivity *in vitro*. In addition to input from other brain areas, interactions with other cell types, such as microglia, astrocytes, oligodendrocytes, or blood arteries descended from mesoderm, affect neural growth and function.

## 5. Applications of brain organoids in modeling Alzheimer’s disease

Alzheimer’s disease (AD) is one neurodegenerative disease that currently cannot be cured by any drug or intervention, due to its complicated pathogenesis. Exploring novel conceptual models of Alzheimer’s Disease (AD) and alternative approaches may lead to the discovery of novel AD treatment options. The creation of efficient disease models, which should ideally replicate all elements of the illness, is the key to understanding AD etiology. Over the years, a variety of methods, including *in vivo*, *in vitro*, and *in silico* platforms, have been used to create disease models of AD (Ranjan et al., 2018). Despite the creation of second generation mouse models that are more advanced and contain humanized sequences, they have often fallen short of accurately recapitulating human AD pathology (Rodriguez-Jimenez et al., 2023).

AD is the most prevalent and devastating neurodegenerative illness with the leading cause of late-onset dementia. More than 46 million people worldwide suffer from dementia, and the projected number of patients is expected to increase to 13.5 million by 2050 as world populations age. Among the most common early clinical symptoms is short-term memory loss, confusion, mood swings, apathy, long-term memory loss, progressive loss of cognition, and disruption of basic functions, such as swallowing, walking, and attention, become increasingly common as AD progresses (Rodriguez-Jimenez et al., 2023). According to Cui et al. (2023), there are few finding on development of AD has been correlated to mitochondrial proteostasis dysfunction regulation in three different aspects which is UPR^mt^, mitochondrial autophagy, and mitochondrial protein import levels (Llanos-González et al., 2020; Cui et al., 2023). Cells damage and cell death or even neuronal death and cognitive impairment regularly caused by dysfunction and homeostasis imbalance of mitochondria as it is the powerhouse of the cells (Li et al., 2023).

Extensive research efforts to understand the complex mechanism of AD in many aspects have confirmed that AD is associated with numerous mutations that can alter gene and neural function (Penney et al., 2020). AD can either be familial (usually early-onset) or sporadic (usually late-onset) in nature. Familial forms of AD (FAD), normally occur within 60–65 years of age, are not very prevalent (< 10 percent), and are inherited in an autosomal-dominant fashion and can be caused by any among over 200 mutations (Ranjan et al., 2018). Recent publication reveals that Apolipoprotein E4 (APOE4) allele remains the strongest known genetic risk factors for late onset AD in which it will manifest abundant alteration of amyloid precursor protein (APP) plaques in beta-amyloid (Aβ) and increase production of cholesterol in astrocytes once the mutation occurs (Ooi et al., 2020; Kulas et al., 2023). The other common gene mutations are Presenilin 1 (PSEN1) and Presenilin 2 (PSEN2), which are also responsible for sequential proteolytic precursors of the APP gene (Delabio et al., 2014; Arber et al., 2020). Subsequently, brain pathology in patients who will eventually develop AD probably begins at least two decades before clinical symptoms appear. Thus, early detection of those at risk of developing AD is essential to diagnose potential treatments for disease change before the existence of harmful neuronal disorders (Butterfield and Halliwell, 2019; Bi et al., 2021).

Thereupon, the advent of iPSCs generated from AD fibroblasts harboring a mutation in APP or PSEN1/2 is one of a way to model AS using iPSCs-based 2-dimensional culture platforms (Ranjan et al., 2018). Given that aging cells is a major limitation for AD and other neurodegenerative diseases, employing stem cells to investigate AD may seem paradoxical. However, since the first features demonstrated that iPSC can be generated using somatic cells, multiple attempts have been made to induce neural stem cells (NSC) and other subtypes such as astrocytes and microglial subsequently (Penney et al., 2020; Zhang et al., 2023). For example, Zhao et al. (2020) proposed a study presenting the generation of iPSC-derived cerebral organoids from AD patients with APOE ε3/ε3or ε4/ε4 genotype which reveals AD patients are associated with an improvement in stress granules and interrupted RNA metabolism. There are also studies using the CRISPR/Cas system that the authors introduced mutations in the APP or PEN1 gene into iPSC, creating an iPSC model for early-onset AD (Paquet et al., 2016). The use of iPSCs to represent illnesses has evolved from employing straightforward culture conditions to more intricate ones. Two-dimensional (2D) cultures, in which developed iPSCs are cultivated as adherent cells on plates, are the norm. However, over the past few years, progress has been made in developing more intricate systems to mimic AD using a combination of 2D (co- or triple-cultures) and 3D (cerebral organoids cultured in suspension, or simply “organoids” cell cultures (Sahlgren Bendtsen and Hall, 2023).

Cerebral organoids made from human induced pluripotent stem cells could simulate NDs in a complex, tissue-like environment that more closely resembles the complexity of the brain in three dimensions (3D). Astrocytes, vasculature, and dysregulated microglia are also known to be present in the parenchyma in AD (Venkataraman et al., 2022; Sahlgren Bendtsen and Hall, 2023). As advancement in the disease modeling using brain organoids continue to improve and becoming increasingly relevant, Table 4 summarized the overview of brain organoids that have been used for Alzheimer’s disease model. In conclusion, generating organoid brain cells such as cerebral or neural derived from iPSC could recapitulate AD-related pathologies, thus solving the puzzles in the pathogenesis of AD.

**Table 4:** Recent studies using organoids derived from iPSC for the modeling Alzheimer’s diseases.

Diseases	Type of starting cells	Gene mutated	Findings	Organoid application	References
Alzheimer’s (AD)	Fibroblast	APOE4	Accelerated Aβ seeding and suppressed Aβ clearance, altered synaptic plasticity.	Human iPSC-organoids recapitulate APOE4-related phenotypes and suggest APOE4-related degenerative pathways that contribute to AD pathogenesis.	Zhao et al. (2020)
	Fibroblast	PSEN1 PSEN2	Reduce Notch signaling and alter neurogenesis.	Premature neurogenesis in fAD iPSCs harboring PSEN1 mutations through cortical differentiation in 2D & cerebral organoid generation in 3D.	Arber et al. (2021)
	Control iPSCs	Mitochondrial peptidase PITRM1	Induces the impairment of mitochondrial proteostasis and the activation of UPR^mt^.	Novel cellular model of human PITRM1 deficiency using human iPSC-derived cortical neurones and cerebral organoids model systems.	Pérez et al. (2021)
	Fibroblast	APP	Aβ accumulation insoluble Aβ aggregation.	Neuroectodermal organoids are used to study the Aβ accumulation implicated in Alzheimer’s disease (AD).	Pavoni et al. (2018)
	Human ES cells (H1) control iPSCs (UE02302)	BACE2	Greater apoptosis and increased levels of Aβ oligomers.	Using human pluripotent stem cell (hPSC)-derived brain organoids to study the expression and functional role of BACE2 in the central nervous system.	Luo et al. (2022)
	Fibroblasts	Cytochrome oxidase (COX) Vmax	Decreased mitochondrial membrane potential, mass, and superoxide generation, as well as decreased mitochondrial respiration parameters.	iPSC derived neurons and cerebral organoids showed reduced COX Vmax in AD subjects.	Flannagan et al. (2022)
	Fibroblasts	ApoE4	Through the induction of beta-secretase 1 (BACE) and glycogen synthase kinase-3 alpha/beta (GSK3ɑ/β) levels, serum exposure raises the levels of ɑ and p-Tau.	Single-cell transcriptomic analysis of brain organoids reveals that serum exposure reduced synaptic function in both neurons and astrocytes and induced immune response in astrocytes.	Chen et al. (2021)
	Fibroblasts	APP PSEN1	It was shown that treating patient-derived organoids with–and–secretase inhibitors greatly lessens the pathology of amyloid and tau.	Detection of significantly higher levels of Aβ in the media from fAD organoids culture compared to controls.	Raja et al. (2016)
	Fibroblasts	PSEN1 Missense mutation (A246E)	In comparison to control cerebral organoids, the relative level of insoluble tau was significantly higher in patients.	Brain organoids created from patients gradually accumulate structures that are very similar to amyloid plaques and neurofibrillary tangles, among other clinical characteristics of AD.	Gonzalez et al. (2018)

## 6. The advantages and limitations in modeling neurodegenerative disease with organoids from iPSCs

iPSC lines from the patients specific cells can be used to model human genetic heterogeneity, but if there is no appropriate control, this effect can confuse disease modeling (Hung et al., 2017; Doss and Sachinidis, 2019). Absence of proper isogenic controls that share single genetic background weakens assumption regarding causal effect of the mutation alone on observed phenotype (Hinz et al., 2019). For instances, the isogenic control cell line from an Alzheimer’s disease patient iPSC line carrying a mutation of A79V in PSEN1 has been edited with the CRISPR/Cas9 system by replacing the point mutation “T” with the wild-type nucleotide “C” thus serves as valuable references to study pathological cellular phenotypes for this mutation (Pires et al., 2016). Biological iPSC lines derived from well-characterized pre-existing iPSC lines of healthy control people that use gene editing techniques can essentially avoid difficulties related to cell line differences. In human embryonic stem cells (ESC) and iPSC, zinc finger nuclei, transcription activator-like effect nuclei (TALENS), clusters of paired nine-based genetics and base genetic editing (cytidine deaminase to convert cytidine to uridine without breaking double strands of DNA) improve genetic editing efficiency due to its relatively simple use and high efficiency compared to other standard technologies.

However, one of the main disadvantages of these gene editing techniques is the possibility of side effects. With Next Gene sequencing technologies, it is possible to investigate potential effects. Although each ESC line has its own clonal difference, iPSC lines are more diverse than ESCs due to their genetic memory, genetic background, and characteristics acquired during reprogramming and differentiation. In addition, incomplete programming due to heterogeneity in differentiation capacity (Efrat, 2021), low proliferation and differentiation potentials causes by aberrant replications at specific region or locus in genome DNA (Paniza et al., 2020), and thus linked to higher genomic DNA methylation (Zhou et al., 2013) are also observed in several iPSC lines despite of their germ-line chimeric abilities. In order to select the completely reprogramed “*bona fide*” iPSC line, evidence-based criteria must be developed.

**Figure 5:**
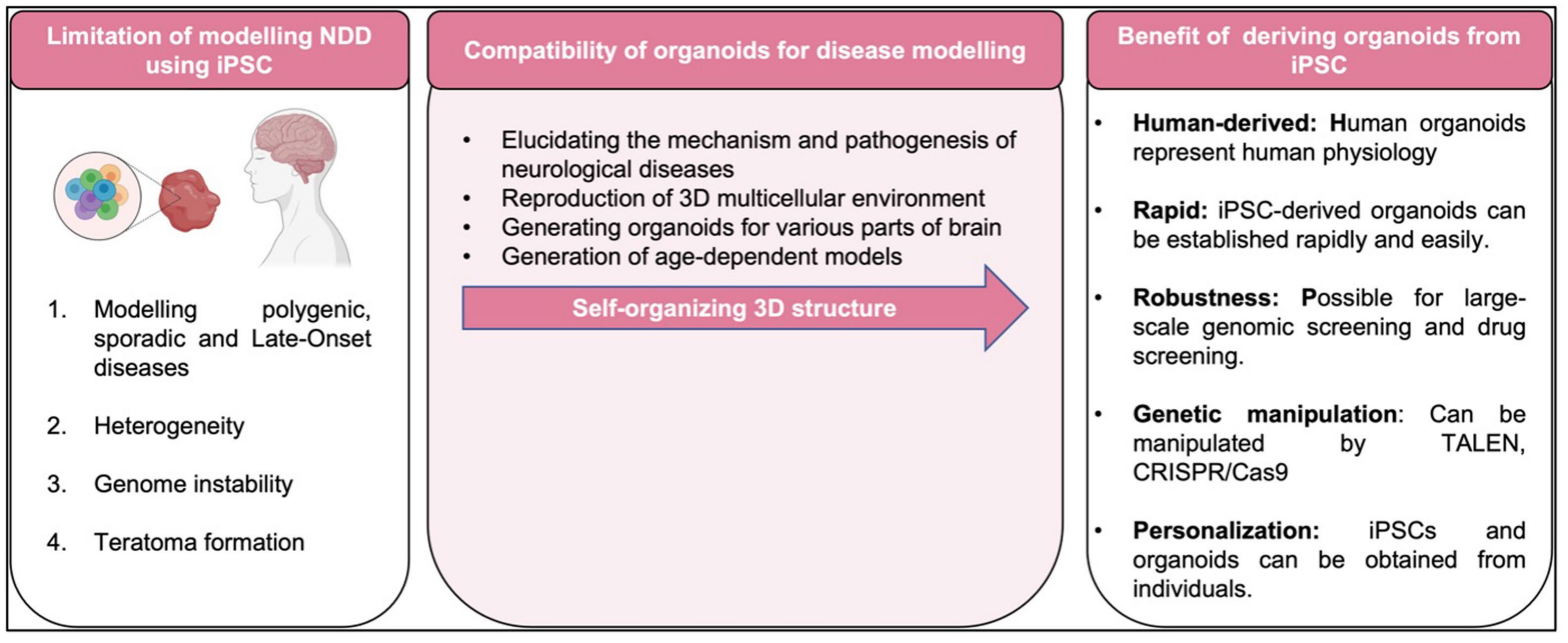
Limitation, compatibility of organoids and benefit of disease modeling with iPSC-derived organoid.

To date, most studies in human iPSCs are concerned with its susceptibility to genetic instability (Zhang et al., 2018), potentially accumulate of chromosome abnormalities (Thompson et al., 2020), large number of copy number variations in early passage iPSC with gradually loss of mosaicism (Yoshihara et al., 2017), and significant shift in the differentiation potential of iPSCs over increasing passage or prolonged iPSC culture (Galiakberova et al., 2023) as summarized in Figure 5. As matter of fact, these challenges pose a challenge to the integrity of iPSC derivatives and the modeling of diseases affected by environmental factors. Therefore, adequate measures should be taken to eliminate cell abnormalities and genetically modified cells that can undermine the integrity of experimental data such as conducting genetic profiles of the cells prior to transplantation and assess the prognostic genetic anomalies that possibly occurred (Yamamoto et al., 2022).

Moving forward to the use of brain organoids, numerous protocols for generating brain organoids have been published which mainly aim to establish the disease modeling on brain disorder. For biomedical study in general and in particular on neural development, regeneration, and pathology of the central nervous system, brain organoids have been a fast growing field (Hoppe et al., 2023). One of them is the neural organs produced from human PSCs, which serves as a platform for studying brain tumors. Normally, progenitor cells are transduced with vectors to introduce mutations that induce brain organoids containing nerve stems, resulting in overgrowth of transduced cells. Thus, comparing to the 2D culture system, brain organoids have improved our understanding of the pathological physiology of neurological diseases (Rowe and Daley, 2019; Sun et al., 2021).

However, despite the drawbacks of brain organoid systems, which are difficult to grow, labor- and money-intensive, lack a vascularization and blood circulation system which may cause the supply for oxygen and nutrients become restricted thus inducing necrosis in the center part of organoids (García-Delgado et al., 2022; Xu et al., 2023) have complex physiological contexts of the intact human brain, and need to be addressed. Recent research has shown that endothelial cells can construct vascular networks in brain organoids, but their functioning is limited because neurons and their progenitors have high metabolic demands, limiting the size of brain organoids to a few millimeters (Hoppe et al., 2023). Organoids can exchange nutrients and waste to the point where they transcend the limitations of diffusion. In order to provide cells less exposed to culture medium with a means for nutrient or waste exchange, researchers have attempted to mimic vascular tissues in response to these difficulties. This is possible in human/animal chimera models, for example by fusing rat vasculature with a human brain organoid, with varying degrees of potential success (AlFatah Mansour et al., 2018; Schneider et al., 2023).

## 7. Future perspectives and conclusions

Human iPSC technology has never seen such a high level of possibility to model any type of cell and hence the state of the disease. The use of advanced stem cell models and an in-depth understanding of the benefits and limitations of the models are probably the best options to move forward, which emphasizes the importance of scientific collaboration because the current limitations of technology are significant but not insurmountable. These restrictions can also be used to imitate other complex diseases, such as heart disease and malignant diseases. As the ability to produce more accurate, subtype-specific cells with correct transcript and epigenome characteristics improves, it will improve the ability to effectively model diseases in order to clarify pathological mechanisms and establish therapeutic applications for patient-derived iPSCs. The parallel development of more complex and physiologically relevant neural tissue models offers exciting opportunities for future models of neuronal shape and function.

Despite massive global investments in brain research, new therapeutic approaches and effective treatments for central nervous system diseases are still lacking, even though the incidence of neurological diseases, mental illnesses and addiction in recent decades has increased considerably due to the changes in population and lifestyle. The recent development of monoclonal antibodies is an important step in the treatment of Alzheimer’s, but much work remains to be done (Quirion, 2023). Numerous findings suggest that cerebral organoids, one type of brain organoid, may be a great model for genetic forms of Alzheimer’s disease, allowing researchers to study the pathology at more physiological levels of gene expression in cortical tissue derived from stem cells with the same genetic background as the patients. Organoids can be produced easily and in huge quantities, and when combined with the adaptability of *in vitro* studies, they may make it possible to examine the underlying disease mechanisms and test the efficacy of potential medication candidates (Gonzalez et al., 2018).

## Author contributions

FN, GT, and WW: conceptualization and visualization and supervision. GT and WW: validation. AJ: writing-original draft preparation. AJ and DS: drawing of figures. FN: project administration and funding acquisition. All authors have read and agreed to the published version of the manuscript.

## Funding

This study supported by the Ministry of Higher Education Malaysia (MOHE) through the award grant FRGS/1/2020/SKK06/UKM/03/4 and the Faculty of Medicine Research Grant [FF-2023-189] from UKM.

## Conflict of interest

The authors declare that the research was conducted in the absence of any commercial or financial relationship that could be construed as a potential conflict of interest.

## Publisher’s note

All claims expressed in this article are solely those of the authors and do not necessarily represent those of their affiliated organizations, or those of the publisher, the editors and the reviewers. Any product that may be evaluated in this article, or claim that may be made by its manufacturer, is not guaranteed or endorsed by the publisher.
